# Supplementary material for: Schizophrenia more employable than depression? Language-based artificial intelligence model ratings for employability of psychiatric diagnoses and somatic and healthy controls
Source: PLoS One. 2025 Jan 8;20(1):e0315768. doi: 10.1371/journal.pone.0315768 (PMC11709238; doi:10.1371/journal.pone.0315768)
Supplement: S1 File — (DOCX) [file pone.0315768.s001.docx]

**Supporting Information 1:** Further results from analogies investigating employability and associated attributes for people with mental health disorders

**S1 Box, Employability of Anxiety Disorder**

-------------------------------------------------------------------------------------------------

*Analogy:“healthy (A) is to employable (B) as anxiety disorder (C) is to _ (D)?”*

*Top 1 returned item: D = psychiatric_disorder*

*Vector Similarity = 0.46184382*

*Top 10 returned items, in descending order:*

[('psychiatric_disorder', 0.46184382),

(' *em*ployable', 0.44487655),

('depressive_episode', 0.44185305),

('personality_disorder', 0.43955797),

('depressive_disorder', 0.42953604),

('mental_disorders', 0.42821088),

('mental_illness', 0.4177483),

('mental_illnesses', 0.4157204),

('dysthymia', 0.4136898),

('depressive_illness', 0.41040692)]

-------------------------------------------------------------------------------------------------

**S2 Box, Reliability of Psychosis**

-------------------------------------------------------------------------------------------------

*Analogy: “healthy (A) is to reliable (B) as psychosis (C) is to _ (D)?”*

*Top 1 returned item: D =* paranoid_delusions

*Vector Similarity=* 0.43029052

*Top 10 returned items, in descending order:*

[('paranoid_delusions', 0.43029052),

('unreliability', 0.4265383),

('psychotic_symptoms', 0.42366505),

('schizophrenia', 0.40502602),

('unreliable', 0.39983946),

('psychotic_disorders', 0.39759386),

('Although_gulfshorelife.com_strives', 0.3940553),

('most_accourate', 0.3917009),

('psychotic', 0.38703436),

('mental_illness', 0.38511735)]

-------------------------------------------------------------------------------------------------

**S3 Box, Reliability of Depression**

-------------------------------------------------------------------------------------------------

*Analogy: “healthy (A) is to reliable (B) as depression (C) is to _ (D)?”*

*Top 1 returned item: D =* manic_depression*,*

*Vector Similarity=* 0.41612798

*Top 10 returned items, in descending order:*

[('manic_depression', 0.41612798),

('depressive_illness', 0.4146071),

('Transcranial_magnetic_stimulation', 0.4077782),

('posttraumatic_stress_disorder_PTSD', 0.39887315),

('bipolar_disorder', 0.39610547),

('manic_depressive_disorder', 0.39318466),

('bipolar_depression', 0.39027578),

('mental_illness', 0.389395),

('undergone_electroshock_therapy', 0.38776484),

('alcoholism', 0.3856067)]

-------------------------------------------------------------------------------------------------

**S4 Box, Reliability of Anxiety Disorder**

-------------------------------------------------------------------------------------------------

*Analogy: “healthy (A) is to reliable (B) as anxiety disorder (C) is to _ (D)?”*

*Top 1 returned item: D =* Although_gulfshorelife.com_strives*,*

*Vector Similarity=* 0.41597575

*Top 10 returned items, in descending order:*

[('Although_gulfshorelife.com_strives', 0.41597575),

('most_accourate', 0.3976212),

('unreliable', 0.3898004),

('EPLRS', 0.37426648),

('personality_disorder', 0.37231058),

('installations_Versant', 0.3706699),

('Denali_PureSpec_verification', 0.36904114),

('unreliability', 0.35606086),

('psychiatric_disorder', 0.35601115),

('Aonix_delivers', 0.35573855)]

-------------------------------------------------------------------------------------------------

**S5 Box, Competency of Psychosis**

-------------------------------------------------------------------------------------------------

*Analogy: “healthy (A) is to competent (B) as psychosis (C) is to _ (D)?”*

*Top 1 returned item: D =* psychotic

*Vector Similarity=* 0.5280237

*Top 10 returned items, in descending order:*

[('psychotic', 0.5280237),

('narcissistic_delusion', 0.49548477),

('schizophrenia', 0.48838636),

('mental_illness', 0.48130852),

('paranoid_schizophrenia', 0.48045614),

('paranoid_schizophrenic', 0.47831684),

('psychotic_symptoms', 0.47770798),

('psychiatrists', 0.47291386),

('borderline_personality_disorder', 0.4710107),

('psychotic_tendencies', 0.47091132)]

-------------------------------------------------------------------------------------------------

**S6 Box, Competency of Depression**

-------------------------------------------------------------------------------------------------

*Analogy: “healthy (A) is to competent (B) as depression (C) is to _ (D)?”*

*Top 1 returned item: D =* mental illness

*Vector Similarity=* 0.4991551

*Top 10 returned items, in descending order:*

[('mental_illness', 0.4991551),

('depressive_illness', 0.47815123),

('psychiatrists', 0.4770743),

('psychosis', 0.47461295),

('bipolar_disorder', 0.4718555),

('manic_depressive_disorder', 0.46618122),

('manic_depression', 0.46574795),

('bi_polar_disorder', 0.46414733),

('narcissistic_personality_disorder', 0.460495),

('schizoaffective_disorder', 0.4580021)]

-------------------------------------------------------------------------------------------------

**S7 Box, Competency of Anxiety Disorder**

-------------------------------------------------------------------------------------------------

*Analogy: “healthy (A) is to competent (B) as anxiety disorder (C) is to _ (D)?”*

*Top1 returned item: D =* personality disorder

*Vector Similarity=* 0.48479766

*Top 10 returned items, in descending order:*

[('personality_disorder', 0.48479766),

('depressive_disorder', 0.4555056),

('schizoaffective_disorder', 0.45102498),

('psychiatric_disorder', 0.4457148),

('schizoid_personality_disorder', 0.4362935),

('narcissistic_personality_disorder', 0.43485302),

('mental_illness', 0.4311293),

('narcissistic_delusion', 0.42854375),

('manic_depressive_disorder', 0.4247742),

('depressive_illness', 0.42371157)]

-------------------------------------------------------------------------------------------------

**S8 Box, Resilience of Psychosis**

-------------------------------------------------------------------------------------------------

*Analogy: “healthy (A) is to resilient (B) as psychosis (C) is to _ (D)?”*

*Top1 returned item: D =* psychotic_symptoms

*Vector Similarity=* 0.45691934

*Top 10 returned items, in descending order:*

[('psychotic_symptoms', 0.45691934),

('psychotic_episodes', 0.4494602),

('paranoid_psychosis', 0.43770438),

('mental_illness', 0.43730628),

('psychotic', 0.4339078),

('schizophrenia', 0.43387994),

('psychoses', 0.4278791),

('psychotic_disorders', 0.4259656),

('psychotic_illness', 0.41834795),

('schizophrenic', 0.41754872)]

-------------------------------------------------------------------------------------------------

**S9 Box, Resilience of Depression**

-------------------------------------------------------------------------------------------------

*Analogy: “healthy (A) is to resilient (B) as depression (C) is to _ (D)?”*

*Top 1 returned item: D =* undergone_electroshock_therapy

*Vector Similarity=* 0.4845298

*Top 10 returned items, in descending order:*

[('undergone_electroshock_therapy', 0.4845298),

('Depression', 0.4414804),

('depression_anxiety', 0.43800622),

('depressive_illness', 0.43176192),

('manic_depression', 0.42997813),

('depressive', 0.42992526),

('childhood_traumas', 0.42945185),

('mental_illness', 0.42389572),

('posttraumatic_stress', 0.42064756),

('bi_polar_disorder', 0.41220805)]

-------------------------------------------------------------------------------------------------

**S10 Box, Resilience of Anxiety Disorder**

-------------------------------------------------------------------------------------------------

*Analogy: “healthy (A) is to resilient (B) as anxiety disorder (C) is to _ (D)?”*

*Top 1 returned item: D =* personality_disorder

*Vector Similarity=* 0.41174597

*Top 10 returned items, in descending order:*

[('personality_disorder', 0.41174597),

('agoraphobia', 0.38915712),

('depressive_illness', 0.37102962),

('schizotypal_personality_disorder', 0.36800206),

('depression', 0.36156166),

('flashbacks_nightmares_sudden', 0.35911098),

('resilience', 0.35832512),

('selective_mutism', 0.3571924),

('manic_depressive_disorder', 0.35577095),

('Asperger_syndrome', 0.35504743)]

-------------------------------------------------------------------------------------------------

**S11 Box, Dentistry and Psychosis**

-------------------------------------------------------------------------------------------------

*Analogy: “healthy (A) is to dentist (B) as psychosis (C) is to _ (D)?”*

*Top 1 returned item: D =* psychiatrist

*Vector Similarity=* 0.5559195

*Top 10 returned items, in descending order:*

[('psychiatrist', 0.5354526),

('orthodontist', 0.49263045),

('periodontist', 0.44760656),

('optometrist', 0.44397986),

('gynecologist', 0.44011834),

('doctor', 0.4333089),

('psychotherapist', 0.4331208),

('oral_surgeon', 0.43269435),

('Dentist', 0.4304645),

('psychologist', 0.42884922)]

-------------------------------------------------------------------------------------------------

**S12 Box, Engineering and Psychosis**

-------------------------------------------------------------------------------------------------

*Analogy: “healthy (A) is to engineer (B) as psychosis (C) is to _ (D)?”*

*Top 1 returned item: D =* mechanical_engineer

*Vector Similarity=* 0.48880413

*Top 10 returned items, in descending order:*

[('mechanical_engineer', 0.48880413),

('electrical_engineer', 0.47324193),

('Engineer', 0.4462324),

('paranoid_delusions', 0.44612715),

('aeronautical_engineer', 0.4340852),

('psychiatrist', 0.4283855),

('engineering', 0.4134974),

('forensic_psychologist', 0.40883088),

('draftsman', 0.40757585),

('geologist', 0.40148526)]

-------------------------------------------------------------------------------------------------

**S13 Box, Finance and Psychosis**

-------------------------------------------------------------------------------------------------

*Analogy: “healthy (A) is to banker (B) as psychosis (C) is to _ (D)?”*

*Top 1 returned item: D =* psychiatrist

*Vector Similarity=* 0.44280943

*Top 10 returned items, in descending order:*

[('psychiatrist', 0.44280943),

('financier', 0.43202308),

('paranoid_delusions', 0.4256023),

('forensic_psychologist', 0.4220062),

('investment_banker', 0.40964484),

('stockbroker', 0.40142012),

('bankers', 0.3998442),

('schizophrenia', 0.3898598),

('Psychiatrist', 0.38902298),

('psychotherapist', 0.38673133)]

-------------------------------------------------------------------------------------------------

**S14 Box, CEO and Psychosis**

-------------------------------------------------------------------------------------------------

*Analogy: “healthy (A) is to ceo (B) as psychosis (C) is to _ (D)?”*

*Top 1 returned item: D =* CEO

*Vector Similarity=* 0.42122337

*Top 10 returned items, in descending order:*

[('CEO', 0.42122337),

('managing_director', 0.40074697),

('chief_executive', 0.39714924),

('psychotic_symptoms', 0.3829317),

('psychotic_episodes', 0.38127923),

('CTO', 0.37864667),

('hallucination', 0.37855005),

('président_directeur_général', 0.37566036),

('paranoid_delusions', 0.37439966),

('COO', 0.37006974)]

-------------------------------------------------------------------------------------------------

**S15 Box, CFO and Psychosis**

-------------------------------------------------------------------------------------------------

*Analogy: “healthy (A) is to cfo (B) as psychosis (C) is to _ (D)?”*

*Top 1 returned item: D =* président_directeur_général

*Vector Similarity=* 0.41958305

*Top 10 returned items, in descending order:*

[('président_directeur_général', 0.44479054),

('zegt', 0.42173517),

('qui_avait', 0.4061371),

('dans_ses', 0.40178287),

('tutti_gli', 0.40156943),

('été_nommé', 0.4012593),

('entendu', 0.39841732),

('M.Sc._P.Eng', 0.39812046),

('jg', 0.3974454),

('comunicado', 0.3972081)]

-------------------------------------------------------------------------------------------------
