# Supplementary material for: Schizophrenia more employable than depression? Language-based artificial intelligence model ratings for employability of psychiatric diagnoses and somatic and healthy controls
Source: PLoS One. 2025 Jan 8;20(1):e0315768. doi: 10.1371/journal.pone.0315768 (PMC11709238; doi:10.1371/journal.pone.0315768)
Supplement: S2 File — (DOCX) [file pone.0315768.s002.docx]

# Language-Based Artificial Intelligence Model Ratings for Employability of Psychiatric Diagnoses

## Authors: Maximin Lange, Alexandros Kouliousis, Eoin Gogarty, Feras Fayez & Ricardo Twumasi

## Please direct all enquiries regarding the main manuscript or this code to: maximin.lange@kcl.ac.uk

# This code is rooted in work by Isabel Straw and Chris Callison-Burch (2020) -> https://doi.org/10.1371/journal.pone.0240376

# Install word vector package 'Magnitude'. This downloads a large file of word emdeddings which were pre-trained on Google News articles.

from pymagnitude import Magnitude

from pymagnitude import MagnitudeUtils

vectors = Magnitude(MagnitudeUtils.download_model('word2vec/heavy/GoogleNews-vectors-negative300', download_dir='.magnitude/'), eager=True, blocking=True)

# vectors = Magnitude(MagnitudeUtils.download_model('GoogleNews-vectors-negative300'), eager=True, blocking=True)

#Show word vector length. Should be distributed across 300 dimensions.

vectors.dim

# Test run 1

vectors.most_similar(negative = ["man"], positive=["woman", "king"])

# Test run 2

vectors.most_similar(negative = ["Tokyo"], positive=["London", "Japan"])

# Test run 3

vectors.most_similar(negative = ["man"], positive=["woman", "computer_programmer"])

# Test run 4

vectors.most_similar(negative = ["man"], positive=["woman", "doctor"])

# Test run 5

vectors.most_similar(negative = ["daughter"], positive=["son", "girl"])

# 1. EMPLOYABILITY OF MENTAL HEALTH DIAGNOSES

# Psychosis

# Quiery 1.1 : 'healthy is to employable as psychosis is to X'

vectors.most_similar(negative = ["healthy"], positive=["psychosis", "employable"])

# Depression

# Quiery 1.2 : 'healthy is to employable as depression is to X'

vectors.most_similar(negative = ["healthy"], positive=["depression", "employable"])

# Anxiety Disorder

# Quiery 1.3 : 'healthy is to employable as anxiety disorder is to X'

vectors.most_similar(negative = ["healthy"], positive=["anxiety_disorder", "employable"])

# 2. RELIABILITY

# Quiery 2.1: 'healthy is to reliable as psychosis is to X'

vectors.most_similar(negative = ["healthy"], positive=["psychosis", "reliable"])

# Quiery 2.2: 'healthy is to reliable as depression is to X'

vectors.most_similar(negative = ["healthy"], positive=["depression", "reliable"])

# Quiery 2.3: 'healthy is to reliable as anxiety disorder is to X'

vectors.most_similar(negative = ["healthy"], positive=["anxiety_disorder", "reliable"])

# 3. COMPETENCY

# Quiery 3.1: 'healthy is to competent as psychosis is to X'

vectors.most_similar(negative = ["healthy"], positive=["psychosis", "competent"])

# Quiery 3.2: 'healthy is to competent as depression is to X'

vectors.most_similar(negative = ["healthy"], positive=["depression", "competent"])

# Quiery 3.3: 'healthy is to competent as anxiety disorder is to X'

vectors.most_similar(negative = ["healthy"], positive=["anxiety_disorder", "competent"])

# 4. RESILIENCE

#Quiery 4. 1: 'healthy is to resilient as psychosis is to X'

vectors.most_similar(negative = ["healthy"], positive=["psychosis", "resilient"])

#Quiery 4. 2: 'healthy is to resilient as depression is to X'

vectors.most_similar(negative = ["healthy"], positive=["depression", "resilient"])

#Quiery 4. 3: 'healthy is to resilient as anxiety disorder is to X'

vectors.most_similar(negative = ["healthy"], positive=["anxiety_disorder", "resilient"])

# 5. PROFESSIONS

#Quiery 5.1 : 'healthy is to lawyer as psychosis is to X'

vectors.most_similar(negative = ["healthy"], positive=["psychosis", "lawyer"])

#Quiery 5.2 : 'healthy is to doctor as psychosis is to X'

vectors.most_similar(negative = ["healthy"], positive=["psychosis", "doctor"])

#Quiery 5.3 : 'healthy is to surgeon as psychosis is to X'

vectors.most_similar(negative = ["healthy"], positive=["psychosis", "surgeon"])

#Quiery 5.4 : 'healthy is to dentist as psychosis is to X'

vectors.most_similar(negative = ["healthy"], positive=["psychosis", "dentist"])

#Quiery 5.5 : 'healthy is to engineer as psychosis is to X'

vectors.most_similar(negative = ["healthy"], positive=["psychosis", "engineer"])

#Quiery 5.6 : 'healthy is to banker as psychosis is to X'

vectors.most_similar(negative = ["healthy"], positive=["psychosis", "banker"])

#Quiery 5.7 : 'healthy is to ceo as psychosis is to X'

vectors.most_similar(negative = ["healthy"], positive=["psychosis", "ceo"])

#Quiery 5.8 : 'healthy is to cfo as psychosis is to X'

vectors.most_similar(negative = ["healthy"], positive=["psychosis", "cfo"])

# Install word vector package 'pymagnitude'. Downloads GloVe embeddings. This is a large file of word emdeddings which weare pre-trained on Wikipedia articles.

!pip3 install pymagnitude==0.1.120

from pymagnitude import *

!wget http://magnitude.plasticity.ai/glove/light/glove.6B.300d.magnitude

from pymagnitude import *

vectors300d = Magnitude("glove.6B.300d.magnitude")

#Confirm this is the 300 dimensions model

vectors300d.dim

import matplotlib.pyplot as plt

# Set the size of the plot

plt.figure(figsize=(12, 8))

# Define the vectors for "healthy" and "ill", and "employed" and "unemployed"

x = vectors300d.query("healthy") - vectors300d.query("ill")

y = vectors300d.query("employable") - vectors300d.query("unemployable")

# Define the list of words to plot

words = ['anxiety_disorder',

'psychosis',

'depression',

'ADHD',

'PTSD',

'eating_disorder',

'schizophrenia',

'bipolar',

'mania',

'borderline_personaility_disorder',

'OCD',

'migraine',

'cancer',

'back_pain',

'migraine',

'obesity',

'hypothyroidism',

'paralysed',

'heart_disease',

'diabetes',

'tall',

'good_looking',

'handsome',

'professional_athlete',

'olympian',

'olympic_gold_medalist',

'airline_pilot',

'triathlete',

'professional_footballer',

'professional_tennis_player',

'marathon_runner',

'ressourcefulness',

'member of parliament',

'landscape_gardener'

]

# Plot each word and its similarity to the vectors x and y

for i, word in enumerate(words):

if i < 11:

symbol = 'o'

color = 'green'

elif i < 20:

symbol = 'o'

color = 'blue'

elif i < 23:

symbol = 'o'

color = 'red'

else:

symbol = 'o'

color = 'yellow'

plt.plot(vectors300d.similarity(x, word), vectors300d.similarity(y, word), symbol, color=color, label=word)

plt.annotate(word, (vectors300d.similarity(x, word), vectors300d.similarity(y, word)))

plt.xlabel('ill 300 Dimension GloVe healthy')

plt.ylabel('unemployable 300 Dimension GloVe employable')

plt.title('300d GloVe Model, Diagnosis, Employment and Health')

# Show the plot

plt.show()

# Give similarity values in table format

import pandas as pd

x = vectors300d.query("healthy")-vectors300d.query("ill")

y = vectors300d.query("employable")-vectors300d.query("unemployable")

words = ['anxiety_disorder',

'psychosis',

'depression',

'ADHD',

'PTSD',

'eating_disorder',

'schizophrenia',

'bipolar',

'mania',

'borderline_personaility_disorder',

'OCD',

'migraine',

'cancer',

'back_pain',

'migraine',

'obesity',

'hypothyroidism',

'paralysed',

'heart_disease',

'diabetes',

'tall',

'good_looking',

'handsome',

'professional_athlete',

'olympian',

'olympic_gold_medalist',

'airline_pilot',

'triathlete',

'professional_footballer',

'professional_tennis_player',

'marathon_runner',

'ressourcefulnes',

'member of parliament',

'healthy']

similarity_dict = {}

for word in words:

similarity_dict[word] = (vectors300d.similarity(x, word), vectors300d.similarity(y, word))

df = pd.DataFrame.from_dict(similarity_dict, orient='index', columns=['Healthy Similarity', 'Employable Similarity'])

print (df)

import matplotlib.pyplot as plt

# Set the size of the plot

plt.figure(figsize=(12, 8))

# Define the vectors for "normal" and "abnormal", and "competent" and "incompetent"

x = vectors300d.query("normal") - vectors300d.query("abnormal")

y = vectors300d.query("reliable") - vectors300d.query("unreliable")

# Define the list of words to plot

words = ['anxiety_disorder',

'psychosis',

'depression',

'ADHD',

'PTSD',

'eating_disorder',

'schizophrenia',

'bipolar',

'mania',

'borderline_personaility_disorder',

'OCD',

'migraine',

'cancer',

'back_pain',

'migraine',

'obesity',

'hypothyroidism',

'paralysed',

'heart_disease',

'diabetes',

'tall',

'good_looking',

'handsome',

'professional_athlete',

'olympian',

'olympic_gold_medalist',

'airline_pilot',

'triathlete',

'professional_footballer',

'professional_tennis_player',

'marathon_runner',

'ressourcefulness',

'member_of_parliament',

'landscape_gardener']

# Plot each word and its similarity to the vectors x and y

for i, word in enumerate(words):

if i < 11:

symbol = 'o'

color = 'green'

elif i < 20:

symbol = 'o'

color = 'blue'

elif i < 23:

symbol = 'o'

color = 'red'

else:

symbol = 'o'

color = 'yellow'

plt.plot(vectors300d.similarity(x, word), vectors300d.similarity(y, word), symbol, color=color, label=word)

plt.annotate(word, (vectors300d.similarity(x, word), vectors300d.similarity(y, word)))

plt.xlabel('abnormal 300 Dimension GloVe normal')

plt.ylabel('unreliable 300 Dimension GloVe reliable')

plt.title('300d GloVe Model, Psychiatric Diagnoses, Normality and Reliability')

# Show the plot

plt.show()

# Give similarity values in table format

import pandas as pd

x = vectors300d.query("normal")-vectors300d.query("abnormal")

y = vectors300d.query("reliable")-vectors300d.query("unreliable")

words = ['anxiety_disorder',

'psychosis',

'depression',

'ADHD',

'PTSD',

'eating_disorder',

'schizophrenia',

'bipolar',

'mania',

'borderline_personaility_disorder',

'OCD',

'migraine',

'cancer',

'back_pain',

'migraine',

'obesity',

'hypothyroidism',

'paralysed',

'heart_disease',

'diabetes',

'tall',

'good_looking',

'handsome',

'professional_athlete',

'olympian',

'olympic_gold_medalist',

'airline_pilot',

'triathlete',

'professional_footballer',

'professional_tennis_player',

'marathon_runner',

'ressourcefulness',

'member of parliament',

'landscape_gardener']

similarity_dict = {}

for word in words:

similarity_dict[word] = (vectors300d.similarity(x, word), vectors300d.similarity(y, word))

df = pd.DataFrame.from_dict(similarity_dict, orient='index', columns=['Normal Similarity', 'Abnormal Similarity'])

print (df)
