# Supplementary material for: Schizophrenia more employable than depression? Language-based artificial intelligence model ratings for employability of psychiatric diagnoses and somatic and healthy controls
Source: PLoS One. 2025 Jan 8;20(1):e0315768. doi: 10.1371/journal.pone.0315768 (PMC11709238; doi:10.1371/journal.pone.0315768)
Supplement: Attachment — Submitted filename: Response to Reviewers.docx [file pone.0315768.s003.docx]

**Rebuttal Letter**

**Reviewer: The conclusion is maybe too definitive, when there are some clear limitations to the findings noted.**

Answer: Abstract has been amended accordingly.

**Reviewer: The introduction is interesting and covers an increasingly important topic. I think it is lacking in some focus around the key topic of employability in psychiatric diagnoses. Suggest including more information around this, perhaps some statistics showing objectively that this is the case.**

Answer: We appreciate this comment and have amended this section accordingly, having added more comprehensive research.

**Reviewer: Very interesting points around how language in CVs can show differences when written by different demographics. Some further examples of this (…)**

Answer: Further examples have been included in the addressed section

**Reviewer: (continued from last query) and some examples of how this has been shown to be the case in psychiatric conditions would be beneficial for the paper.**

Answer: Thank you for pointing out this important distinction, which we didn’t make clear in the paper. We did not find any investigations into writing style of people with mental health disorders (this would be an excellent idea for a future paper). What we argue, instead is the following: Specific word use patterns of people with mental illness have been identified, while detection of general mental illnesses and even reliable estimation of population mental health is possible through analyses of social media posts, surveys, narrative writings, and interviews. At the same time, deep learning models can detect social media users at risk for developing a mental disorder, deduced from online posts represented with linguistic features at different levels, including messages and corresponding writing style and emotions conveyed. Hence, even though no study has looked at resume writing by people with mental health disorders, we argue, the chances of an NLP algorithm being able to detect differences between healthy people and people with mental health disorders would be extremely high, especially with the current speed of change within NLP techniques. We have rewritten the relevant paragraph in the paper to address this point.

**Reviewer: Quite casually written in places – could perhaps be restructured to a more academic style. E.g. the sentence “Similarly, on the other end of the job searching stick, job openings had to be looked up manually by individuals in the market for a new position. Especially online, options could be endless.”.**

Answer: Amended

**Reviewer Sentence structure can be difficult to read where sentences are particularly lengthy – section 1.2, paragraph 3 has 5 commas. Consider splitting up to multiple sentences in areas like this.**

Answer: Amended

**Reviewer: t-SNE is not explained. Please provide this in core text.**

Answer: t-SNE is already explained in section 2.1, third paragraph. However, if the reviewer wishes further explanation we will add it. We further added a short sentence to the beginning of section 3.2.1.1.

**Reviewer: Results are exhaustive and can be difficult to follow in places. Suggest improving presentation of this section – perhaps using tables or similar to present the analogies and vector results more succinctly. Could some be migrated to supplementary information so only the core questions are included?**

Answer: Fair suggestion – we did migrate most of the findings to the supplementary material and only left core questions included.

**Reviewer: More emphasis needed around comment that these results are not in agreement with other research in this area. Why is this the case?**

Answer: We believe we have already offered sufficient explanation for this in the paragraphs six to eight in the discussion section of the paper. We dedicated two paragraphs to the differences in our methodology (e.g., Straw and Callison-Burch (2020) forced a biased return) and one paragraph to our hypothesis that differences might be due to reporting in the news about mental health has become more positive and liberating in recent years, which might contribute to lesser associations of mental health disorder and stigma related to employability mirrored in word embeddings.

**Reviewer: Notably, the authors state that vector results past 1 may not be accurate as is seen in the test analogy London is to England as Tokyo is to ____. The vector scores in this instance are quite close to the top scoring response. Should these be written off across the board or perhaps the method isn’t functioning as expected and a different approach to measure this should be taken? Without context on how writing in CVs of those with psychiatric disorders may differ from those without, it’s difficult to judge whether this is the more appropriate approach to analysing this. It doesn’t seem as though it can be definitively stated that certain psychiatric conditions are viewed less favourably than physical conditions. The analogy method, though a useful exercise in building the foundations of how conditions are viewed relative to healthy controls, a more systematic and informed approach is possibly needed to unravel this question.**

Answer: We use the same method, perhaps even better executed, as Straw and Callison-Burch (2020; since our model is not forcing a biased return). Claiming the method isn’t functioning as expected and a different approach to measure this should be taken, would render Straw & Callison-Burch’s paper redundant as well.

There remains no gold-standard or rule for evaluating analogy returns. Nissim et al., (2020) suggest considering top-5 up and sometimes the top-10 returned items. It is furthermore expected that accuracy would decrease, the further one goes away from the top- 1. We do, however, note an interesting point: When querying the reverse, i.e. Tokyo is to Japan as London is to X, the model gives more sensible answers.

This might speak for the fact that more is being written about London and the UK (in English) than there is about Tokyo and Japan (the word2vec we investigate was trained on Google News articles) thus resulting in those words featuring more in the training text corpus, resulting in more accurate embeddings. In the same notion, there might be more written about depression, however less about schizophrenia, thus resulting in more embeddings for depression, thus leaving more room for discrimination as they are being picked up more frequently.

This would leave embeddings for schizophrenia be less accurate, however, at the same time, less discriminating. Individuals with schizophrenia would therefore, by accident due to fewer text and resulting word embeddings, be discriminated less.

Above we already argued for the fact that we could not find in the literature any investigations into writing style of people with mental health disorders. Still, specific word use patterns of people with mental illness have been identified. Hence, even though no study has looked at resume writing by people with mental health disorders, we argue, given the evidence presented in the earlier paragraph, the chances of an NLP algorithm being able to detect differences between healthy people and people with mental health disorders would be high.

Of course, as we acknowledge in Section 2.5.1 as well as 4.2, there are more sophisticated measures than word analogies, but, like we argue in the mentioned section, we still believe in validity and relevance of our methodology.

**Reviewer : Minor comments:
Last sentence in final paragraph of 1.1 Background has two full stops.
Second last paragraph in section 2.4. is missing an apostrophe.
Second last line of section 3.2.2. there are some font size inconsistencies.
Second line of second paragraph in section 4.2. there is highlighted text where there shouldn’t be.**

Answer: All have been amended.
