# Supplementary material for: Schizophrenia more employable than depression? Language-based artificial intelligence model ratings for employability of psychiatric diagnoses and somatic and healthy controls
Source: PLoS One. 2025 Jan 8;20(1):e0315768. doi: 10.1371/journal.pone.0315768 (PMC11709238; doi:10.1371/journal.pone.0315768)
Supplement: Attachment — Submitted filename: Response to Reviewers 2.docx [file pone.0315768.s004.docx]

**Rebuttal Letter**

**Journal Requirements**

**Editor: Please review your reference list to ensure that it is complete and correct. If you have cited papers that have been retracted, please include the rationale for doing so in the manuscript text, or remove these references and replace them with relevant current references.**

Answer: Is there a chance the editor could confirm the retracted reference they are referring to? After inspection of the list, we could not find a retracted article. We suspect that reference no. 17 in the last version of the manuscript ([10.1093/schbul/sbn024](https://doi.org/10.1093%2Fschbul%2Fsbn024)) might have been a suspect, as there is a paper from the same lab, closely related and discussing similar findings (10.1080/09540260802564516) which indeed has been retracted, but which we never cited. If there is a different reference that has been retracted, please let us know and we will remove it. In any case, we removed reference no. 17 ( [10.1093/schbul/sbn024](https://doi.org/10.1093%2Fschbul%2Fsbn024)) due to comments by reviewer #3, more on that in the direct response to the reviewers.

**Editor: In the methods section, you describe the use of Word2Vec and GloVe algorithms to assess bias in NLP models concerning employability. Can you clarify if these models were retrained with any specific modifications or if they were used as pre-trained models directly?**

Answer: The models were used as they were, directly downloaded from 'word2vec/heavy/GoogleNews-vectors-negative300', download_dir='.magnitude/' and

<http://magnitude.plasticity.ai/glove/light/glove.6B.300d.magnitude>

with no re-training. We added a sentence regarding this information in the sections ‘2.3 Word2Vec’ and ‘2.4 Global Vectors for Word Representation (GloVe)’

**Editor: Furthermore, how did you ensure that the analogy tasks and cosine similarity measures were sufficiently sensitive and specific to detect subtle forms of bias that might not be overt?**

Answer: Word analogies and cosine similarity have indeed limitations in detecting complex biases, and there are more sophisticated methods to detect subtle forms of bias. This has been discussed in our section ‘2.5.1 Alternative Methods’. However, these methods were chosen for their established use in bias research and their interpretability in our specific context, as well as building on work by Straw and Callison-Burch (2020). As mentioned in our section ‘4.2 Future research’ while our results show limited overt bias, we recognize the need for more nuanced techniques like WEAT or SEAT to uncover subtle biases. Future work will explore these methods to build upon our findings. More on this in our response to reviewer #4.

**Editor: The results section provides a detailed account of findings from both Word2Vec and GloVe embeddings. However, the discussion around these findings seems somewhat brief considering the complexity of the data. Could you expand on the potential societal implications of these biases in AI models? For instance, how might these biases influence real-world HR practices if these AI models are employed without correction?**

Answer: We have covered, at length, the topic of how these biases influence real-world HR practices if these AI models are employed without correction in our sections “1.3.1 Recruiting and Occupational Bias in NLP” and “1.3.2 Job Recommender Systems and Occupational Bias in NLP”. We do not see the need to elaborate on this in the discussion.

**Reviewers**

**Reviewer #3: The results suggest that in language analysis, there is not a strong negative bias against mental health disorders. However, while this is interesting in theory, the authors do not provide sufficient argument as to how this would actually apply in the context of the hiring process. Unless engaged in supported employment, most job seekers with serious mental health conditions do not disclose to employers although employers might be able to discern mental health history from social media searches. Therefore, it is unclear what the results of NLP of mental health disorders and employability would look like in resume parsing. The utility of the results from an analysis of general text data rather than an analysis of resume and employment data analysis needs to be made more strongly especially as the findings may be biased by the general exclusion of people with serious mental health conditions from the workforce.**

Answer: We agree that job seekers with serious mental health conditions often do not disclose to employers in their resumes. What we are saying is: Our findings should make job seekers with mental health disorders hopeful, as they *could* openly disclose their condition to employers *without* facing discrimination. We have added this information to our conclusion.

Of course this might be the result of not enough people disclosing it, which in turn results in fewer mentioning in the initial corpus and thus limited discrimination, i.e. there is a chance this lack of discrimination is not a result of changing attitude towards mental health disorders on the side of employers, but rather, as discussed in this and the last revision round as well as in our discussion section, it just might be the lack of mentioning in the training corpus. This would still not change the fact that there appears to be low bias.

**Reviewer #3**: **The summary of the literature on employment of people with serious mental illnesses shows some misunderstandings. Stigma is a barrier to employers hiring people with SMI, but not necessarily a barrier to wanting to work. Desire to work among adults with SMI in spite of individual, interpersonal, and structural barriers has been shown extensively. In addition, Supported Employment requires competitive employment as an outcome by definition; the two are not in opposition in the paper cited.**

Answer: That is true. We amended the corresponding paragraph.

**Reviewer #3: What is the evidence that people with disclosed or undisclosed psychiatric diagnoses would not fall into the desired category of mentally healthy? Evidence of social group identification from NLP has been given for race and gender categories, but not psychiatric disorders except for the studies cited which used self-disclosed diagnoses as an identifier. In the absence of identification of mental health disorders either through self-disclosure or language analysis, this logic remains only theoretical. This weakens the argument for the utility of the study.**

Answer: This is a very similar argument to a query raised by reviewer #1 in the previous round. As discussed last time, and as reviewer #3 points out correctly, there are no investigations into writing style of people with mental health disorders (this would be an excellent idea for a future paper).

What we argue, instead, is the following: Specific word use patterns of people with mental illness have been identified, while detection of general mental illnesses and even reliable estimation of population mental health is possible through analyses of social media posts, surveys, narrative writings, and interviews. At the same time, deep learning models can detect social media users at risk for developing a mental disorder, deduced from online posts represented with linguistic features at different levels, including messages and corresponding writing style and emotions conveyed.

Contrary to what reviewer #3 states, the studies cited (predominantly those from our section ‘1.3.1 Recruiting and Occupational Bias in NLP’ ) do *not* use self-disclosed diagnoses as an identifier.

Hence, even though no study has looked at resume writing by people with mental health disorders, we argue, the chances of an NLP algorithm being able to detect differences in writing styles between healthy people and people with mental health disorders would be extremely high.

**Reviewer #3: To say that people with mental health disorders “could still perform the job well” and might be excluded “perhaps even with good reason” is opinion and biased language, as is the assumption that non-western or “non-conventionally written” resumes would be associated with mental illness. Similarly, use of the word “normal” and “normality” as the contrast to mental illness is problematic and exclusionary. While a contrast needs to be made, the word “normal” for people without a disability is not inclusive language. While a contrast needs to be made, the word “normal” for people without a disability is not inclusive language.**

Answer: We apologise for this oversight; we have now followed American Psychological Association guidelines to refer to people experiencing mental illness with bias free language.

We have amended the paragraph, changing ‘normal’ to ‘healthy’.

We did not state that we assume non-western or non-conventionally written resumes would be associated with mental illness. We state that NLP algorithms trained on data of healthy individuals, when encountering a non-western or non-conventionally written resume or cover letter, might *mistakenly* assume these resumes as written by people experiencing mental illness, only because it is different to the training data. This is speaking to the un-inclusive manner of training data going into algorithms, not to an association between non-western writing style and mental illness. Hence, we called it a false-positive scenario for exclusion, i.e. the algorithm mistaking anything that is not what it was trained on gets automatically excluded.

**Reviewer #3: It is encouraging that in the analysis, there was not a strong negative association between mental health conditions and professions. However, it’s not clear that this absence of association in language would actually translate to less bias in resume parsing or employment. This lack of bias may reflect the profound exclusion of people with psychiatric disabilities from the labor force, rather than greater employability within the labor force. In other words, because people with psychiatric disabilities are generally excluded from employment, there would be less word embeddings of the two. This would also explain the finding that “more severe mental health disorders are more employable… than common mental health disorders” given that more common mental health disorders may occur in conjunction with employment more frequently in the data. Could this unexpected finding also be an artifact of the role of employed professionals in diagnosing and reporting on serious mental illness?**

Answer: This is a very good point, which, however, had been addressed in our discussion.

This paper was not an investigation into employability of people with mental health disorders, hence our findings do not comment on employability of this group in the labour force. Our findings only reflect how the word2vec algorithm estimates the employability of people with mental health disorders. As, at the moment, they seem to not be discriminated by the word2vec algorithm, they might be encouraged to openly disclose their condition. We addressed the point that, because people with psychiatric disabilities are generally excluded from employment, there would be less word embeddings of the two, in our discussion section. This, however, does not change the fact that the algorithm appears to hold low bias against these individuals. The algorithm might thus do the right thing for the wrong reason.

What is true is a potential scenario as follows:

Supposedly more people disclose their mental health disorder, still get hired. Algorithms would be newly trained on data featuring individuals with mental health disorders. There are two extreme scenarios: Either they perform just as good, or even better than their health peers, which would then in turn lead to even less discrimination in the algorithm.

The second option would be people with mental health disorders performing worse than healthy people, thus getting excluded from the workforce, thus resulting periodically in an algorithm that has learned to exclude mentally ill people. However, after some time, through their consequent absence in the training data, when retraining the algorithm again at a later point in time, they are again not featured in the training corpus, i.e. the algorithm again has low bias against these individuals and the cycle starts again. This seems to be the point reviewer #3 tries to make.

We do not know what will happen, only that in both cases, the algorithm seems to not discriminate against people with mental health disorders, if potentially for the wrong reasons.

**Reviewer #4: One reviewer commented about the presentation of the results from the Word2Vec analogies, which has now been transformed into several tables. Nonetheless, it appears lazy, selective, and is hard to digest as a reader in this manner. I would therefore suggest that the authors perhaps combine analogies and top 5 or top 10 results into bigger tables with rows for the results and columns for the analogies (e.g. one table for professions, one table for employability attributes...).**

Answer: As reviewer #4 states here, another reviewer in the previous revision round already gave us feedback on our method of results presentation, with which we complied. This new suggestion now seems to be a personal style preference and we believe our method of presentation as it currently stands, as done in compliance with the earlier reviewer, is sufficient in presenting our results in a clear and understandable way for a reader.

**Reviewer #4: Additionally, I struggle with the assertion made here "Word embeddings thus deliver a stable, reliable and valid estimate of biases." Aside from the fact that this strong conclusion follows a series of weak arguments with a lot of hedging ("often", "the notion", "can reflect"), it is later argued in the paper that word embeddings are (as most people in the field know) only as good at representing the world as the data they have been trained on and schizophrenia might show less bias because it is underrepresented in the data compared to depression (a good point to make). So how valid, reliable, and stable is this estimate?**

Answers:

Regarding the notion that a strong conclusion follows a series of weak arguments: We do not follow the reasoning that the arguments are weak. We refuted and disproved arguments and methodological flaws by other researchers, who misinterpreted earlier research.

Regarding the notion that there is a lot of hedging: We do not see a problem with that.

Regarding the notion that it is later argued in the paper that word embeddings are (as most people in the field know) only as good at representing the world as the data they have been trained on and schizophrenia might show less bias because it is underrepresented in the data compared to depression: As we keep saying in our rebuttal letters, we do not question that there are likely few mentions of schizophrenia in the training data and thus probably inaccurate word embeddings as a result. Still, the model does not discriminate against schizophrenia.

Regarding the question ‘So how valid, reliable, and stable is this estimate?’: See query above

**Reviewer #4: The argument given for using word embeddings and GLoVE rather than more sophisticated quantitative methods ("an open-ended analogy approach allows for a wider scope of discovery") appears weak in light of not finding biases.**

Answer: The absence of detected biases in our results should not be interpreted as a weakness of the method, but rather indicative of the possible characteristics of the underlying data, which may suggest a lack of overt discriminatory bias within the corpus used. Denouncing our method as weak because no bias was found is similar to calling a blood test inaccurate only because it does not find disease within a healthy patient. Future enhancements, as outlined, will include more nuanced methods such as WEAT, further refining our ability to detect and interpret subtle biases in employment-related contexts concerning mental health.

**Reviewer #4: Finally, two things struck me in the discussion on GLoVE embeddings. I'm by no means an expert on t-SNE and only have a vague notion on what the algorithm does, but I was wondering how sensible it is to compare the relative positions of any two points in this reduced space. I've always understood it more as a basic visualization technique that attempts to maintain clusters, so to what extent can two points be meaningfully compared? I may be off, but for me, the clusters that you colored green/blue/yellow/red are more intuitively represented in the space than the individual** **points**.

Answer: The reviewer raises a valid point regarding the fact that this visualisation technique is most reliable for understanding the overall clustering and grouping of the data points, and that the main utility of t-SNE visualisations is to get an overall sense of the clustering and grouping of the high-dimensional data points, rather than making precise inferences about individual point positions or distances. However, what we are demonstrating here, unlike the reviewer suggests, clusters that we have colored green/blue/yellow/red are *not* intuitively represented. Our point is: there are no clear-cut clusters. We have added a sentence regarding this point to our discussion section. Furthermore, comparing individual data points has been done by Straw and Callison-Burch (2020), whose methodology we followed closely and who were published in your journal.

**Reviewer #4: Additionally, in this part of the discussion some word choices are made that are usually associated with statistical analyses while they seem to be made here on the basis of visual inspection (e.g., "marginally"). Perhaps consider phrasing this more clearly.**

Answer: In this instance, the word “marginally” was used to qualitatively describe subtle variations observed through visual inspection of data plots, rather than to denote statistically significant differences. We recognise the potential for confusion, and we agree that a clearer phrasing could enhance the manuscript’s precision and readability.

To address your concern, we propose to replace “marginally” with “slightly” to avoid any connotation of statistical analysis and to better convey that the observed differences are minimal and noted primarily through graphical interpretation. This modification will be applied throughout the manuscript to ensure consistency and clarity in how we communicate observations derived from visual data assessments.

**Reviewer #4: Spelling of author Nissim is incorrect in some places (Nisism) and at least once I've spotted psychosis as "psychois."**

Answer: All amended.
