# Supplementary material for: Schizophrenia more employable than depression? Language-based artificial intelligence model ratings for employability of psychiatric diagnoses and somatic and healthy controls
Source: PLoS One. 2025 Jan 8;20(1):e0315768. doi: 10.1371/journal.pone.0315768 (PMC11709238; doi:10.1371/journal.pone.0315768)
Supplement: Attachment — Submitted filename: Response to Reviewers 4.docx [file pone.0315768.s005.docx]

**Rebuttal Letter**

**Journal Requirements**

**Editor: Please review your reference list to ensure that it is complete and correct. If you have cited papers that have been retracted, please include the rationale for doing so in the manuscript text, or remove these references and replace them with relevant current references.**

Answer: As stated in our last rebuttal letter that featured this exact comment, we kindly ask the editor to confirm the retracted and/or missing reference they are referring to, since, after inspection of our list, we could not find a retracted article and we believe our reference list is complete and correct.

**Reviewers**

**Reviewer #4: I still find it difficult to draw conclusions from [the presentation of the result] and while my suggestion may be personal and does absolutely not have to be followed in that format (it was just a suggestion to save the authors time), I do invite the authors to reconsider their presentation because I stand by my original comment that it appears lazy (no further explanation or directing of attention in text, only printed tables that are never referenced in the text), selective (why these tables and not others that were moved to supplementary materials?), and is not very useful to the reader in this manner (what are you trying to get the reader to see with this selection? and are you achieving it?).**

Answer:

The reviewer makes four different arguments regarding presentation of results, specifically the tables.

First, ***“****no further explanation or directing of attention in text, only printed tables that are never referenced in the text”.*

We have now added text in the result section referencing to all tables used in that section.

Second, the reviewer thinks our presentation of the results appears “*selective (why these tables and not others that were moved to supplementary materials?”.*

In earlier versions that were submitted to the journal we included all tables. However, reviewer #1 advised us to move some to the supplementary material. We do accept the critique that it appears selective. There is no order to the inclusion of the findings, or to selection of which went to the supplementary material. This is since they all investigate very similar queries (mental health and employability). Further, none but one analogy table featured bias in the top-1 returned word. We included that table in the main manuscript. We added text regarding this in the newly added text in the results section.

Third, the reviewer critiques our presentation of the results to be “*not very useful to the reader in this manner (what are you trying to get the reader to see with this selection, and are you achieving it?”*

We added new text to the methods section 2.3 Word2Vec, stating that we select job titles and fields that are associated with prestige and status, e.g., medicine, law, engineering, finance. Competition to enter such occupations is hence higher than most jobs. This, we argue, would result in easily detectable potential bias against mental illness, is often aimed at ability. We are showing the reader that in the word embeddings examined, analogies did not contain biased words within the top-10 returned items, indicating that the model might see people with mental illness as equally able to enter and/or perform in high earning professions. We have added a statement explaining this in the results section.

The reviewer is further alluding to a comment from the last round of reviews, suggesting to “*combine analogies and top 5 or top 10 results into bigger tables with rows for the results and columns for the analogies”*.

Doing this, we believe, would complicate reading and understanding of the reader as then they would be bombarded with large tables of over 10 analogies.

We hope we have found a middle ground with the suggestions of the reviewer and our own views regarding presentation of the results. We appreciate the continuous feedback and commitment to the improvement of this manuscript by all reviewers.
